# Die Corona-Pandemie – eine Katastrophe mit Sprengkraft

**DOI:** 10.1007/s11609-020-00416-4

**Published:** 2020-11-23

**Authors:** Klaus Dörre

**Affiliations:** Institut für Soziologie, Friedrich-Schiller-Universität Jena, Carl-Zeiß-Straße 3, 07743 Jena, Deutschland

**Keywords:** COVID-19, Pandemie, Zangenkrise, Bonapartismus, Globalisierung, Klimagerechtigkeit, Öffentliche Soziologie, COVID-19, Pandemic, Pincer-grip crisis, Bonapartism, Globalization, Climate justice, Public Sociology, COVID-19, Pandémie, Double crise, Bonapartisme, Mondialisation, Justice climatique, Sociologie publique

## Abstract

Die Corona-Pandemie ist eine medizinische Katastrophe, die sich auf eigentümliche Weise mit einer epochalen ökonomisch-ökologischen Zangenkrise verbindet – so lautet die Kernthese des Beitrages. COVID-19 wird als „äußerer Stoß“ definiert, dem eine tiefe Rezession folgt. Sieht man von der natürlichen Virenmutation ab, lassen sich Pandemie, Rezession und Zangenkrise als unterscheidbare Repulsionen einer Hyperglobalisierung verstehen, die sukzessive ihre eigenen Voraussetzungen untergräbt. Ohne den Finanzcrash von 2007 bis 2009, das politische Interregnum der Nachkrisenjahre und die Tendenz zu bonapartistischen Demokratien lässt sich die neuerliche Zäsur nicht begreifen. Spontan führt die Corona-Krise keineswegs zu einem „build back better“. Der Staat des Ausnahmezustands ist zu solchen Weichenstellungen kaum in der Lage. Stattdessen wächst die Gefahr, dass harte Verteilungskämpfe, zunehmende Ungleichheit und Entsolidarisierung eine Nachhaltigkeitswende zusätzlich erschweren.

## Abstract (en)

The corona pandemic is a medical catastrophe that is intricately intertwined with an epochal economic-ecological double or “pincer-grip” crisis – thus goes the central thesis of this contribution. COVID-19 is defined as an “external shock” followed by a deep recession. Leaving aside the natural process of viral mutation, the pandemic, recession and pincer-grip crisis can be understood as distinct repulsions of an hyperglobalization that is gradually undermining its own conditions of existence. Meanwhile, the recent rupture cannot be adequately grasped without taking into account the financial crash of 2007 to 2009, the political interregnum of the post-crisis years and the tendency towards bonapartistic democracy. By itself, the corona crisis will not lead to a “build back better”; the emergency state is hardly capable of such a setting of the course. Instead, there is a rising danger that fierce conflicts over distribution, increasing inequality and desolidarization will make a turn to sustainability even harder.

## Résumé

La thèse centrale de cet article est que la pandémie de coronavirus est une catastrophe sanitaire qui se conjugue de manière singulière à une double crise économique et écologique d’une portée historique. Le COVID-19 est défini comme un « choc externe » suivi d’une profonde récession. Abstraction faite des mutations naturelles à l’origine du virus, la pandémie, la récession et la double crise peuvent être conçues comme différents phénomènes de répulsion d’une hyper-mondialisation qui sape progressivement ses propres conditions d’existence. Cette nouvelle césure ne peut être comprise indépendamment du krach financier de 2007 à 2009, de l’interrègne politique des années qui ont suivi et de la tendance favorable aux démocraties bonapartistes. D’elle même, la crise du coronavirus ne conduit aucunement à une « reconstruction mais en mieux » (*build back better*). L’état d’urgence n’est guère en mesure de poser les jalons d’une telle reconstruction. À la place, le risque grandit que des luttes pour le partage des richesses, des inégalités croissantes et une désolidarisation ne compliquent encore la transition vers la durabilité.

## Stimmungswechsel

„So viel Wissen über unser Nichtwissen und über den Zwang, unter Unsicherheit zu handeln und leben zu müssen, gab es nie“, konstatiert Jürgen Habermas angesichts der Corona-Pandemie und empfiehlt den Sozialwissenschaften Zurückhaltung anstelle „unvorsichtiger Prognosen“ (Habermas 2020). Er hat recht. Über SARS-CoV‑2 weiß die Fachwelt noch immer wenig. Sicher ist nur: Das Virus wirkt aggressiv, kann töten und ist trotz zwischenzeitlicher Lockerung von Abstandsregeln auch in Europa noch keineswegs besiegt. Daten zu Infizierten und bereits Gestorbenen sind hochgradig ungenau. Ob und wann ein wirksamer Impfstoff in ausreichenden Mengen hergestellt werden kann, vermag niemand präzise vorherzusagen. Das Krisenmanagement lässt sich erst mit zeitlichem Abstand angemessen beurteilen. Wie die Rezession, die der Pandemie folgt, verlaufen wird, ist ebenfalls unklar.

Bei den wirtschaftlichen Verwerfungen zeichnet sich immerhin eine eindeutige Tendenz ab. Der Internationale Währungsfonds spricht von einer „Jahrhundertkrise“ (IMF 2020). Tatsächlich übertrifft der Einbruch im ersten Halbjahr 2020 selbst den des Finanzcrashs von 2007–2009. Allein die US-Wirtschaft schrumpfte im zweiten Quartal 2020 um 9,5 %. Bis Juni 2020 wurden im Land offiziell 44,2 Mio. Anträge auf Arbeitslosenhilfe registriert. Bereits zu Beginn der Pandemie waren 81 % der „global workforce“ (circa 2,7 Mrd. Menschen) vom Lockdown ganz oder teilweise betroffen. Als besonders verwundbar erweisen sich informell und prekär Arbeitende sowie die Belegschaften kleinerer Unternehmen (ILO 2020). Auch die deutsche Ökonomie ist hart getroffen. Im Vergleich zum vorigen Quartal ging die Wirtschaftsleistung im zweiten Quartal 2020 preis-, saison- und kalenderbereinigt um 9,7 % zurück (–11,3 % im Vergleich zum Vorjahresquartal; Destatis 2020). Einen größeren Einbruch des Bruttoinlandsprodukts (BIP) hat es in der Bunderepublik nie gegeben. Für etwa zwölf Millionen Menschen war bis zum Juni 2020 Kurzarbeit angemeldet; real arbeiteten sechs bis sieben Millionen Beschäftigte in reduzierter Zeit. Obwohl die Talsohle des konjunkturellen Abschwungs wohl durchschritten ist, spricht wenig für einen raschen Aufschwung. Jedes fünfte Unternehmen fürchtet um seine Existenz (Ifo Institut 2020). Wichtige Konzerne planen einen großvolumigen Stellenabbau, zugleich geht die Angst vor einer Insolvenzwelle um. Folgerichtig macht sich Ernüchterung breit. Habe es anfangs Menschen gegeben, die „verzaubert von der ‚Entschleunigung‘ der Gesellschaft“ sprachen, werde gerade „etwas sehr stark entschleunigt“ (Fromm und Hägler 2020).

Man ahnt, was folgen könnte. Je länger Pandemie und Rezession andauern, desto wahrscheinlicher wird, dass das optimistische „build back better“ der Anfangsphase verhallt. Wer sich Stimmungsschwankungen nicht ausliefern will, ist deshalb gut beraten, erst einmal eigene Diagnosen zu früheren Krisen selbstkritisch zu hinterfragen. Das soll nachfolgend geschehen. Soziologisch betrachtet ist die Corona-Pandemie eine medizinische Katastrophe, ein „äußerer Stoß“ (Braudel 1986, S. 720), der sich, so die These, auf eigentümliche Weise mit einer epochalen ökonomisch-ökologischen Zangenkrise verbindet. COVID-19 trifft auf eine hochgradig verflochtene Weltwirtschaft, in der eine Viruserkrankung zum ökonomischen Zusammenbruch führen kann. Wegen der außergewöhnlich schweren Rezession wird es für die verwundbarsten Teile der Weltbevölkerung um das nackte Überleben gehen. Massive Entsolidarisierungen könnten die Folge sein. Käme es dazu, würde zusätzlich erschwert, was längst überfällig ist: eine weltweite Wende zugunsten ökologischer und sozialer Nachhaltigkeit. Der Beitrag wendet sich daher gegen eine quasi-religiöse Krisensemantik, der zufolge „ein geglückteres soziales Sein“ (Steil 1993, S. 10) als Chance auch in der Corona-Pandemie liege (Abschnitt 2). Argumentiert wird, dass ein politisches Interregnum die Wende zur Nachhaltigkeit blockiert hat (Abschnitt 3). Mit dem Ausnahmestaat, der die Pandemie managt, haben die Kräfte des politischen Zentrums in wichtigen europäischen Ländern die Initiative zurückgewonnen. Ihr Staatsinterventionismus reibt sich indes an sozialen Verwerfungen und soziobiologischen Ordnungen von langer Dauer, die einer Nachhaltigkeitswende entgegenstehen (Abschnitte 4 und 5). Mit den Sustainable Development Goals (SDG) der Vereinten Nationen verfügt eine öffentliche Soziologie der Nachhaltigkeit immerhin über einen Maßstab, um das Erreichte am eigentlich Nötigen zu messen (Abschnitt 6).

## Welche Krise, welche Lösungen?

Beginnen wir mit der Finanzkrise von 2007–2009, deren schwere Nachbeben noch immer spürbar sind. Wie Joris Steg (2020, S. 73) kritisch anmerkt, hatte ich diese Kontraktion frühzeitig als „große Transformationskrise“ (Dörre 2009, S. 70) charakterisiert und sie auf eine Stufe mit der Großen Depression (1873–1895), der Großen Weltwirtschaftskrise (1929–1932) und der Neuen Depression (1973–1974) gestellt. Eine solche Krise, so meine damalige Überlegung, würde systemimmanent nur im Zuge einer „Revolution-Restauration“ (Gramsci 1991, S. 1362) zu bewältigen sein. Gemeint war ein Typus sozialen Wandels, der, ohne Masterplan und strategisches Zentrum, alle sozialen Verhältnisse umwälzt, aber die Kernstruktur kapitalistischer Produktionsweisen bewahrt. Eine wirtschaftsdemokratische Transformation des Kapitalismus als wünschbare Vision vor Augen, hielt ich einen Green New Deal für die erreichbare politische Option. Als einer von vielen hatte der US-Ökonom James K. Galbraith dem soeben gewählten US-Präsidenten Barack Obama zu einem solchen Programm geraten. Es zielte auf ein Wirtschaftswachstum, das hauptsächlich über öffentliche Investitionen in erneuerbare Energien und in die soziale Infrastruktur erreicht werden sollte (Galbraith 2008). Andere Krisendiagnosen gingen deutlich weiter. Sie prophezeiten ein rasches Ende des Neoliberalismus oder gar einen Niedergang des gesamten kapitalistischen Weltsystems (Wallerstein 2014).

„All diese Annahmen, Erwartungen und Hoffnungen wurden bitter enttäuscht“, stellt Joris Steg (2020, S. 73) im Rückblick fest und zählt mich zu jenen Wissenschaftlern, die mit ihren Prognosen falsch gelegen haben. Damit trifft er einen wichtigen Punkt. Weder existiert eine lineare Steigerungslogik, die abgelöst von politischen Entscheidungen, gesellschaftlichen Machtverhältnissen und periodischen Krisen immer weiter eskaliert, noch findet sich ein Automatismus, der erwünschten Auswegen aus Krisen zum Durchbruch verhelfen könnte.^1^ Große Krisen sind Kreuzpunkte, von denen höchst unterschiedliche Pfade gesellschaftlicher Entwicklung abzweigen können. An solchen Wegscheiden ist die Wiederholung des „Sündenfalls“ einer „Sprengung rein ökonomischer Gesetzmäßigkeiten durch politisches Handeln“ nötig, weil sonst ein wirtschaftlicher Zusammenbruch „unvermeidlich“ wäre (Arendt 2006, S. 335). Für den Übergang zu einer postkapitalistischen Formation müsste noch etwas hinzukommen. Der Kapitalismus „kann nicht durch einen ‚endogenen‘ Verfall zugrunde gehen; nur ein äußerer Stoß von extremer Heftigkeit im Verein mit einer glaubwürdigen Alternative könnte seinen Zusammenbruch bewirken“ (Braudel 1986, S. 720).

Diese Einschätzung des Jahrhunderthistorikers Fernand Braudel gilt uneingeschränkt auch für die Corona-Pandemie und die ihr folgende Wirtschaftskrise. Trotz des heftigen Stoßes, den die Seuche zweifelsohne darstellt, gibt es keinen Corona-Determinismus. Vielmehr schlägt die Stunde der Politik – und zwar auch dann, wenn sie Handlungen unterlässt. Weil das so ist, lohnt ein Blick auf das „lange Jahrzehnt“, das der Pandemie vorausgegangen ist. Exogene Schocks, wie z. B. den von Fukushima 2011, gab es auch während dieser Zeit. In ihren Auswirkungen auf das Alltagsleben wohl nicht ganz so dramatisch wie die Corona-Pandemie, hatte die nukleare Katastrophe globale Folgen und führte zum Ausstieg Deutschlands aus der Kernenergie. Im gleichen Jahr erreichte eine weltweite Revolte gegen den Neoliberalismus, die von so unterschiedlichen Akteuren wie Occupy Wall Street, Podemos und Syriza, Klimaaktivisten und feministisch-antirassistischen Initiativen getragen wurde, ihren Höhepunkt (vgl. Kraushaar 2012). All diese Versuche, die marktradikale Hegemonie durch einen Systemwechsel zu überwinden, sind, an ihren Zielen gemessen, gescheitert.

Die Ursachen können hier nicht einmal ansatzweise aufgearbeitet werden. Ein wichtiger Grund liegt jedoch auf der Hand. Die verschiedenen oppositionellen Bewegungen agierten mit einem unzureichenden Verständnis jenes epochalen Umbruchs, zu dem der Finanzcrash nur der Auftakt war. Die Kontraktion von 2007 bis 2009 ist, hier korrigiere ich meine eigene Auffassung, weit mehr als eine große Krise kapitalistischer Akkumulation. Es handelt sich um einen Wendepunkt in den Gesellschaft-Natur-Beziehungen. Ich habe diese Zäsur als ökonomisch-ökologische Zangenkrise bezeichnet (Dörre 2019, S. 3). Der Begriff besagt, dass das wichtigste Mittel zur Überwindung von Stagnation, Arbeitslosigkeit und Armut sowie zur Pazifizierung von Klassenkonflikten im Kapitalismus, die Generierung von Wirtschaftswachstum, unter Status-quo-Bedingungen (hoher Emissionsausstoß, ressourcen- und energieintensiv sowie auf fossiler Grundlage) ökologisch zunehmend destruktiv und deshalb gesellschaftszerstörend wirkt. *Der Zangengriff von Ökonomie und Ökologie markiert eine Krise, die ungelöst hinter der Corona-Pandemie lauert*. Diese Krise ist keine „normale Anomalie“ (Steg 2020, S. 71). Schon wegen ihrer Komplexität ist sie voraussichtlich von langer Dauer. Nachhaltigkeitsziele, fixiert in den SDGs, auf die sich nahezu die gesamte Staatengemeinschaft verpflichtet hat (UN 2015), klagen jedoch ein, dass die Zangenkrise ein Ende finden muss, wenn nicht große Teile des Planeten unbewohnbar werden sollen. Längst handelt es sich bei diesen Zielen um mehr als nur um unverbindliche Absichtsbekundungen. Mit der sanktionierbaren Festlegung auf eine vollständige Dekarbonisierung der europäischen Wirtschaft bis 2050 hat der Transformationsdruck industrielle Schlüsselbranchen erreicht (Dörre et al. 2020). Nicht nur die früh industrialisierten Staaten, auch die Schwellenländer stehen vor einer Nachhaltigkeitsrevolution, deren Zeitbudgets in dem Maße schrumpfen, wie die anvisierten Veränderungen hinausgezögert werden.

## Was blockiert Veränderungen?

Während des langen Jahrzehnts nach dem Finanzcrash haben sich Beharrungskräfte vor allem im politischen System bemerkbar gemacht. Die Unfähigkeit regierender Mitte-rechts- und Mitte-links-Koalitionen, eine Wende zur Nachhaltigkeit auch nur annähernd in Gang zu setzen, begünstigte die Herausbildung einer besonderen Form der Staatlichkeit, die hier als Tendenz zu bonapartistischen Demokratien bezeichnet wird (vgl. Azzará 2019). Bonapartismus (Marx 1960 [1852]) benennt eine Ausnahmeform (Hall 2014, S. 92) des Staates, die das Spannungsverhältnis von Kapitalismus und Demokratie in einem politischen Interregnum stillstellt. Entsprechende Theoreme betonen die Autonomie des Politischen (Brunkhorst 2018, S. 32; Jessop 2018). Krisen erzeugen demnach lediglich den Problemrohstoff, der in Staatsapparaten und von zivilgesellschaftlichen Akteuren kleingearbeitet werden muss. Als Erklärung wird Bonapartismus immer dann interessant, wenn das Spannungsverhältnis von Kapitalismus und Demokratie offen zutage tritt, ohne dass eine Auflösung der zugrundeliegenden Pattsituation in Sicht wäre. Auch wenn sich einfache Übertragungen historischer Analysen auf die Gegenwart verbieten (vgl. Beck und Stützle 2018), besitzen Bonapartismustheorien noch immer ein beträchtliches Anregungspotenzial. Im Unterschied zu anderen autoritären Herrschaftsformen zeichnet sich die bonapartistische Ausnahmeform durch drei Strukturmerkmale aus: die verhinderte Revolution (1), ein Interregnum, das die Kräfte des Neuen gefangen hält (2), sowie ein „transformismo“, bei dem Teile der subalternen Klassen ihre Interessen mangels Alternativen an autoritäre Führer und Formationen delegieren (3). Alle genannten Strukturmerkmale prägten das Jahrzehnt zwischen globaler Finanzkrise und Corona-Pandemie.

**Figure 1:**
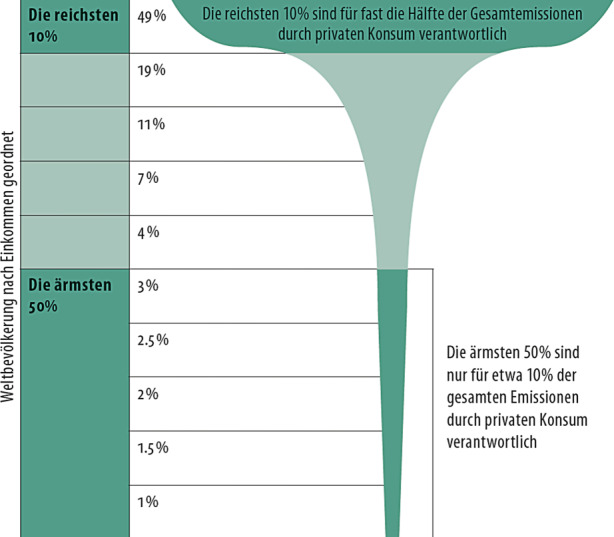

1. *Blockierte Revolution.* Was mit blockierter Nachhaltigkeitsrevolution gemeint ist, lässt sich am Beispiel Klimagerechtigkeit zeigen. Grundsätzlich gilt: Je höher die Einkommen, desto größer der Anteil am Ausstoß klimaschädlicher Emissionen (vgl. Abb. 1). Während das oberste Einkommensdezil der Weltbevölkerung 49 % der konsumbedingten Emissionen verursacht, sind die untersten 50 % gerade einmal für 10 % verantwortlich (Gallagher und Kozul-Wright 2019, S. 22). In Deutschland lagen die durchschnittlichen Pro-Kopf-Emissionen 2019 bei 7,9 Tonnen Kohlenstoffdioxid; einkommensbedingt schwankten die Werte allerdings zwischen fünf und 20 Tonnen. Nötig ist eine Reduktion auf durchschnittlich zwei Tonnen jährlich, das heißt, alle müssen einsparen, aber der Veränderungsdruck ist in den oberen Einkommensklassen besonders groß. Rechnet man nach Unternehmenseinheiten, erzeugen die 20 größten fossilen Produzenten etwa 35 % der globalen Emissionen (Climate Accountability Institute 2019). Ökologische Großrisiken lösen daher keineswegs klassenübergreifende Allbetroffenheit aus, wie Ulrich Beck (1986, S. 7 ff., 48) behauptet hat.^2^ Der Klimawandel betrifft alle, aber eben nicht in gleicher Weise. Werden die unteren Einkommensklassen übermäßig mit den Kosten des ökologischen Umbaus belastet, setzen sie sich, wie etwa in Gestalt der französischen Gelbwesten, zur Wehr. Zielkonflikte zwischen ökonomischer und sozialer Nachhaltigkeit werden so zum Hemmschuh für Veränderungen. Daran zeigt sich, dass der Großkonflikt um eine klimagerechte Ordnung zwischen stark divergierenden Interessengruppen ausgetragen wird. In Anlehnung an Immanuel Wallerstein (Wallerstein 2014, S. 44 f.) lassen sich vier Lager unterscheiden, von denen jeweils zwei entweder dem herrschenden „Geist von Davos“ (Weltwirtschaftsforum) oder dem gegenhegemonialen „Geist von Porto Alegre“ (Weltsozialforum) zugeordnet werden können.
2. *Interregnum.* Für die Pattsituation ist ausschlaggebend, dass die beiden Lager des „Geistes von Porto Alegre“ häufig gegeneinander arbeiteten. Jene Formationen, die eine Kontinuität der alten sozialistischen und Arbeiterbewegungen repräsentieren (vertikale Organisation, Kampf um die Macht), verorteten sich überwiegend auf der Achse des Kapital-Arbeit-Konflikts. Das konkurrierende Lager libertärer Strömungen und Bewegungen setzte hingegen auf Selbstorganisation (funktionale Dezentralisierung), lehnte ökonomisches Wachstum als Ziel emanzipatorischer Politik grundsätzlich ab und agierte neben den Achsen Ethnie/Nationalität und Geschlecht hauptsächlich auf dem Feld des ökologischen Gesellschaftskonflikts. Zahlreiche Spaltungen zwischen diesen beiden Lagern des „Geistes von Porto Alegre“ verhinderten die Herausbildung einer wirkmächtigen politischen Alternative von unten.
  Auf der Gegenseite erleichterte das den gemäßigt-liberalen, globalisierungsaffinen Eliten ein modifiziertes Weiter-so. Die Hauptkräfte dieses dem „Geist von Davos“ verpflichteten Mehrheitslagers, die im politischen Koordinatensystem mitte-rechts und mitte-links angesiedelt sind, näherten sich einander an. Ihre bevorzugten Gegner waren die systemoppositionellen Kräfte in den Lagern des „Geistes von Porto Alegre“. Mit dem Austeritätsdiktat der europäischen Institutionen wurde an Griechenland ein machtpolitisches Exempel statuiert. Ließen sich die Reaktionen auf die Finanzkrise in den USA noch von einer klaren „klassenorientierte[n] Logik“ leiten („Schützt die Wall Street zuerst, um Otto Normalverbraucher kümmern wir uns später“; Tooze 2018, S. 25), glich das europäische Krisenmanagement eher der „Geschichte eines Zugunglücks, eines Wirrwarrs widersprüchlicher Visionen […], des Versagens der Führung und des Versagens kollektiver Aktionen“ (ebd.). Insgesamt schwächte das die Veränderungsbereitschaft des regierenden sozialen Blocks. Ökologische Gefahren ging man primär mit marktkonformen Mitteln an. Zugleich nahmen vertikale Ungleichheiten und soziale Verwundbarkeiten trotz des moderaten Wirtschaftswachstums zu. In einer über Handel, Finanzströme und ausländische Direktinvestitionen zunehmend enger verflochtenen Welt stieg die Verschuldung erneut, während die Investitionen außerhalb des Finanzsektors stagnierten und die gesellschaftliche Infrastruktur bröckelte. Die klimaschädlichen Emissionen kletterten auf ein Rekordniveau; gleichzeitig nahm die Fluchtmigration bei wachsender Weltbevölkerung erheblich zu (vgl. Tab. 1). Autoritätsverluste des politischen Zentrums, aber auch Spaltungen, Niederlagen und Demobilisierungen in den Lagern des „Geistes von Porto Alegre“ blockierten die überfällige Nachhaltigkeitsrevolution und mündeten in ein verlorenes Jahrzehnt.
3. *Transformismo.* Das politische Interregnum begünstigte einen „transformismo“ (Gramsci 1991 ff., S. 98, 101 ff.) des autoritären Lagers. „Transformismo“ bezeichnet die politische Fähigkeit, in Krisensituationen den Bruch mit dem Bestehenden glaubwürdig zu verkörpern, um so als Problemlöser führungsfähig zu werden. Die radikale Rechte präsentierte sich als Protagonistin einer „imaginären Revolte“ (Dörre et al. 2018), deren Protest die Rückkehr zu Verhältnissen einklagte, die nicht wiederherstellbar sind. Auf die Globalisierung antwortete sie mit Neo-Nationalismus, auf Ungleichheit sowie Fluchtmigration mit der Ethnisierung von Verteilungskonflikten und auf den Klimawandel mit dessen Leugnung oder Relativierung. Daraus resultierten politische Polarisierungen, die – wohl vorschnell – als neuer „cleavage“ von Globalisten und Kommunitaristen (vgl. de Wilde et al. 2019) oder als Spaltung zwischen globalisierungsaffinen Anywheres und globalisierungsskeptischen Somewheres gedeutet werden (vgl. Goodhart 2017). Nach meiner Auffassung signalisieren solche Polarisierungen vor allem, dass sich Auseinandersetzungen auf der Klassen- und der Naturachse mehr und mehr zu einem sozial-ökologischen Transformationskonflikt verdichten (Dörre et al. 2020). Nur jene Akteure, die Schlüsselthemen beider Konfliktfelder aufnehmen, haben die Chance, das politische Interregnum zugunsten von Nachhaltigkeitszielen zu überwinden.

**Table 1:** 

	1980	2016
BIP	11,2	76
Bevölkerung (Milliarden)	4,4	7,4
Handel (Exporte)	2,3	20,9
Bestand an ausländischen Direktinvestitionen	0,7	26
Vermögenswerte	12	300
Staatsverschuldung^a^	14	198,6
Migranten (Millionen)	150^b^	250^c^
CO_2_-Emissionen	18 kt	36 kt

^a^Private Unternehmensschulden nicht eingeschlossen

^b^Zahl für 1990

^c^Durchschnitt der Jahre 2015 und 2017

## Worin besteht das Besondere der Corona-Krise?

Auf das Interregnum bonapartistischer Demokratien traf als äußerer Stoß die Corona-Pandemie. Zu unterscheiden sind die natürliche Virenmutation, die Krankheit COVID-19 als medizinische Katastrophe sowie die Wirtschaftskrise als Folgewirkung. Viren sind die kleinsten bekannten Mikroorganismen; sie müssen zellbasierte Lebensformen infizieren, um ihren Lebenszyklus zu vollenden (Wolfe 2020, S. 32). Ob Viren Menschen gefährlich werden, hängt neben ihrem Tötungspotenzial vor allem von ihrer Ausbreitungsfähigkeit ab. Seuchen sind ein „äußerer Stoß“, weil sie im Zuge der Virenmutation ursprünglich außerhalb gesellschaftlicher Funktionsmechanismen entstehen. Daran ändert auch die „tellurische Kraft“ (Antonio Stoppani zit. n. Gerhardt 2020, S. 82), die menschliche Naturwesen zu Verursachern einer erdgeschichtlichen Epochenwende, dem Übergang zum Anthropozän (Crutzen 2019), gemacht hat, im Grunde nichts. Viren entstehen in der außermenschlichen Natur.^3^ Sobald sie menschliche Zellen infizieren und Krankheiten verursachen, werden sie zu einem gesellschaftlichen Problem. Virenerkrankungen breiten sich im „web of life“ (Moore 2015, S. 1) aus; das exogen erzeugte Virus wird endogenisiert. Gesellschaft reproduziert sich dann mit der Viruserkrankung und durch die Pandemie hindurch. Medizinische Versorgung, staatliche Hygiene- und Gesundheitspolitiken sind, wie auch manche Ursachen von Virenübertragungen, immer schon gesellschaftlich geprägt.

Auf Seuchen stoßen wir in allen bekannten Gesellschaftsformationen; sie sind kein spezifisch kapitalistisches Phänomen. Pandemien und Krankheiten wie COVID-19 machen noch immer soziobiologische Ordnungen von langer Dauer sichtbar. Ein bekanntes historisches Bewältigungsmuster von Seuchen folgt dem alten toskanischen Sprichwort: „Keine bessere Arznei gegen die Malaria als ein gut gefüllter Kochtopf“ (Braudel 1985, S. 78). Opfer von Seuchen werden vor allem diejenigen, die aufgrund von Armut und Hunger besonders verwundbar sind. Damit verbunden ist ein anderes Muster, die Entsolidarisierung: „Sobald sich die Seuche ankündigt, brechen die Reichen Hals über Kopf nach ihren Landgütern auf; jeder denkt nur noch an sich: ‚Diese Krankheit macht uns grausamer gegeneinander als Hunde‘“, zitiert Fernand Braudel (ebd., S. 83) einen Zeitzeugen (Samuel Pepys) der Pest in London 1665. Grausamkeit geht mit der Suche nach Sündenböcken einher. Hexenverfolgungen im Spätfeudalismus, die der Schwarzen Pest nachfolgten und der gewaltsamen Unterdrückung häretischer Bewegungen dienten, sind ein markantes Beispiel (Federici 2015).

Beide genannten Bewältigungsmuster hatten über Jahrhunderte hinweg Bestand; gemeinsam mit Hungersnöten regulierten sie den demografischen Wandel. Ihre soziale Verbindlichkeit büßten die überkommenen soziobiologischen Ordnungen erst ein, als es Medizin und staatlicher Gesundheitspolitik gegen Ende des 18. und im Verlauf des 19. Jahrhunderts allmählich gelang, die Seuchen einzudämmen. Der Preis waren anhaltende Spannungen zwischen erfolgreicher Hygienepolitik einerseits und der leichteren Transmission von Krankheitserregern andererseits (Osterhammel 2009, S. 271), die sich auch im 20. Jahrhundert fortsetzten. 1918 starben an einer Grippewelle mehr Menschen als während des gesamten Ersten Weltkriegs.^4^ Daran gemessen ist die Corona-Pandemie eine historische Zäsur, aber kein Ereignis von säkularer Einzigartigkeit.

Eine Besonderheit der Krankheit COVID-19 gegenüber früheren Pandemien besteht im Modus ihrer gesellschaftlichen Endogenisierung. Sieht man von ihrem natürlichen Ursprung in der Virenmutation mit tierischen Wirten ab, kann die Corona-Pandemie durchaus als eine Repulsion intensivierter Globalisierung begriffen werden.^5^ Ein Landnahme-Zyklus, der Verflechtungen zwischen alten und neuen kapitalistischen Zentren primär über expansive Marktbeziehungen hergestellt hat, zehrt zunehmend an den eigenen sozialen Voraussetzungen und kehrt die Globalisierung in einigen ihrer Dimensionen regelrecht um. Einmal endogenisiert, entspricht COVID-19 durchaus diesem Muster. Dass derartige Krankheiten zu Beginn des 21. Jahrhunderts für Menschen zu einer globalen Bedrohung werden können, hängt eng mit der Zunahme weltweiter Reisetätigkeit, der Ausdehnung des internationalen Handels, einem schwindenden Lebensraum für Wildtiere, engeren Mensch-Tier-Kontakten, verbreiteter Massentierhaltung sowie dem Klimawandel und den von ihm ausgelösten Artenwanderungen zusammen. Resistenzen, die eine Behandlung von Virenkrankheiten erschweren, gesellen sich hinzu. Sozialgeografisch begünstigen Regionen mit feucht-warmem Klima die natürliche Virenmutation. Eine solche Krankheit auf die Ursprungsregion zu begrenzen, ist in Zeiten intensivierter Globalisierung nahezu ausgeschlossen. Die Erdteile und ihre ökonomischen Zentren sind trotz aller Ungleichzeitigkeiten so eng miteinander verbunden, dass eine rasche Ausbreitung von Krankheitserregern unvermeidlich ist – und das auch, weil die intensivierte Globalisierung die Zonen sozialer Verwundbarkeit vergrößert hat.

Netzwerke inter- und transnationaler Konzerne beschäftigen, überwiegend indirekt, ein „Weltproletariat“ (Fulcher 2007, S. 125), das mehrheitlich in prekären Verhältnissen oder informell arbeitet. Die Kombination kapitalistischer Produktionsweisen mit patriarchaler Dominanz und rassistischer Abwertung produziert erdteilübergreifend die billigsten Arbeitskräfte. Lohnabhängige in den kapitalistischen Zentren profitieren teilweise von den niedrigen Preisen für Güter, die durch Überausbeutung in den transnationalen Wertschöpfungsketten möglich werden. Aufgrund von Standortwettbewerb und schwindender Gewerkschaftsmacht sind jedoch auch in den früh industrialisierten Ländern des globalen Nordens die Löhne vor allem der Industrie- und Produktionsarbeiter zwischen 2000 und 2013 erheblich gesunken (IMF 2017), während prekäre Arbeits- und Lebensverhältnisse deutlich zugenommen haben (vgl. Schmalz und Sommer 2019; Leite et al. 2020). Die genannten Tendenzen offenbaren die Zwiespältigkeit von Produktions- und Lebensweisen, die auf finanzkapitalistischen Landnahmen beruhen. In den früh industrialisierten Ländern erfolgt die Integration großer Teile der beherrschten Klassen nicht mehr mithilfe des Lohns oder mittels des Kredits, sondern über einen Konsum tiefpreisiger Güter, die in Wertschöpfungsketten und für Märkte hergestellt werden, in denen Überausbeutung sowie rassistische und sexistische Abwertung strukturprägend sind.

Zu diesem System gehört, dass sich mit zunehmender sozialer Verwundbarkeit auch die gesundheitlichen Risiken vergrößern. Daran ist die globale Nahrungsmittelindustrie maßgeblich beteiligt. Die industrielle Herstellung landwirtschaftlicher Produkte bevorteilt Großplantagen, setzt Kleinproduzenten unter Druck, zwingt sie in überwiegend prekäre Lohnarbeit und festigt die Dominanz marktbeherrschender Biotech-Konzerne. Insbesondere die Verhältnisse in der auf Massentierhaltung angewiesenen globalen Fleischindustrie mit ihren unhygienischen Arbeitsbedingungen und der Beschäftigung hochgradig prekärer Werkvertragsarbeiter, die teilweise in Elendsquartieren hausen müssen, bergen gesundheitliche Großrisiken. Weil die gewinngetriebene Kommodifizierung auch vor Saatgut, Pflanzen, Wasser und selbst vor Genen nicht Halt macht, weiten sich die Gefahrenherde aus. Ein expandierender globaler Tourismus, der die erdteilübergreifende Kontakthäufigkeit erhöht, sorgt dafür, dass sich Krankheitserreger rasch in allen Weltregionen ausbreiten können. Deshalb ist es kein Wunder, wenn der Lockdown die für das „Massenschöne“ (Maase 2008) zuständigen Industrien – Reiseveranstalter, Airlines, das Hotel- und Gaststättengewerbe, die gesamte Tourismusbranche, Sportvereine, aber auch Kultureinrichtungen und Konzertagenturen – mit besonderer Härte trifft.

Auf Pandemie und Rezession reagieren zwei Grundvarianten von Staatsaktivitäten, die untereinander nur lose gekoppelt sind. COVID-19 wird von einem Ausnahmestaat bearbeitet, der sich einerseits innerhalb des verfassungsrechtlichen Rahmens bewegt, um andererseits Grundrechte zumindest auf Zeit einzuschränken. Einzige Legitimation für den Ausnahezustand ist die Bekämpfung der Pandemie. Der Staat setzt verbindliche Abstandsregeln durch, die eine rasche Ausbreitung der Seuche unterbinden sollen. Die Regeln und Aktivitäten des rigiden Ausnahmestaats, wie sie in vielen Ländern gelten, müssen sich mit den liberalen Hygiene-Regimes in anderen Ländern messen, die, wie in Schweden, auf einen Lockdown verzichten. Die andere Grundvariante von Staatsaktivitäten ist ein Anti-Rezessions-Interventionismus, der auf Stabilisierung und Wiederaufbau der Wirtschaft zielt. Seine Legitimität resultiert aus der Bekämpfung des Konjunktureinbruchs, der auch jene Staaten heimsucht, die auf die Durchsetzung eines strengen „social distancing“ verzichten.^6^

## Wie werden Pandemie und Rezession bewältigt?

Gibt es Anzeichen dafür, dass das staatliche Krisenmanagement mit soziobiologischen Mustern von langer Dauer bricht, um eine Nachhaltigkeitswende einzuleiten? In einem Punkt lässt sich diese Frage mit einem klaren Ja beantworten. Leben zu retten hat für viele demokratische Regierungen zumindest zeitweilig den Vorrang vor Wirtschaftlichkeitserwägungen erhalten. Obwohl ein Primat der Gesundheit den Nachhaltigkeitszielen der UN entspricht („Goal 3: Ensure healthy lives and promote well-being for all at all ages“), ist das alles andere als selbstverständlich. Die Bilder mit den Leichenbergen aus der Lombardei ließen europäischen Regierungen, die demokratischer Legitimation bedürfen, trotz anfänglichen Zögerns wohl keine andere Wahl. Mit dem Management der Wirtschaftskrise verhält es sich ähnlich. Als „symmetrischer Schock“ hat die Corona-Pandemie Auswirkungen sowohl auf die Angebots- als auch auf die Nachfrageseite. Staaten, die es sich finanziell leisten können, reagieren mit aufwendigen Hilfs- und Konjunkturprogrammen. Dabei haben sie aus der Kontraktion von 2007–2009 gelernt. Von der schwarzen Null in öffentlichen Haushalten bis zur Schuldenbremse werden Leitprinzipien marktradikaler Ökonomik zumindest zeitweilig und ohne nennenswerten Widerstand ad acta gelegt. Jahrzehntelang abgewertet und unterbezahlt, findet die Systemrelevanz von Pflege- und Erziehungsberufen öffentliche Anerkennung. Schlachthöfe, immer wieder vergeblich als Hort von menschenunwürdigen Arbeitsbedingungen angeklagt, müssen künftig mit dem Verbot von Werkverträgen und tiefen Eingriffen in ihr Geschäftsmodell rechnen.

Mit dem neuen Staatsinterventionismus, so jedenfalls die Signale im öffentlichen Raum, wird das Interregnum des „verlorenen Jahrzehnts“ überwunden. Der „transformismo“ wandert, jedenfalls in der Bundesrepublik und einigen anderen europäischen Staaten, wieder in die politische Mitte. Bei wichtigen Repräsentanten wirkt es geradezu, als befreie sie das Virus aus der Gefangenschaft verkrusteter Strukturen und Denkschablonen. Das „build back better“, ursprünglich eine Strategie der Vereinten Nationen zur Förderung von Nachhaltigkeit nach Katastrophen, ist im Eliten-Talk jedenfalls allgegenwärtig: „Wir haben jetzt die Gelegenheit, unser gesamtes Wirtschaftsmodell kritisch zu überprüfen und die Exzesse der Globalisierung da zu korrigieren, wo sie zu den dramatischen Auswirkungen der Pandemie beigetragen haben. Deshalb sollten wir bei der Wiederbelebung unserer Wirtschaftssysteme besonderes Gewicht auf deren soziale und ökologische Nachhaltigkeit legen“, gibt Wolfgang Schäuble, Vordenker im liberal-konservativen Lager des „Geistes von Davos“, die Orientierung vor (Schäuble 2020). Schäuble plädiert für einen europäischen Green Deal, Mindestpreise für CO_2_, ein mutigeres Voranschreiten bei der europäischen Integration und eine gemeinsame europäische Armee mit der Fähigkeit zum Krieg. Der so proklamierten Veränderungsbereitschaft haben derzeit weder die autoritären Elitenfraktionen noch die Kräfte des „Geistes von Porto Alegre“ Gleichwertiges entgegenzusetzen.^7^

### Die Pandemie als Ungleichheitsverstärker

Ein bekundeter „transformismo“ der konservativen Mitte ist das eine, die empirisch fassbare Realität etwas anderes. Schaut man genauer hin, wird sichtbar, dass die altbekannten Muster der Seuchenbewältigung keineswegs völlig verschwunden sind. Auch COVID-19 schlägt bevorzugt dort zu, wo im übertragenen Sinne der volle Teller Suppe fehlt. Weil es einen vollständigen Stillstand der Ökonomie nie gegeben hat, wirkte das Krisenmanagement vom ersten Tag an als Ungleichheitsverstärker. Konnten viele Angestellte ins Homeoffice wechseln, mussten gewerblich Arbeitende häufig auch dann in die Fabrik, wenn es dort an elementaren Schutzvorkehrungen fehlte. Die millionenfach verordnete Kurzarbeit ist mit erheblichen Einkommensverlusten für die Betroffenen verbunden. Nur 54 % der Beschäftigten von Betrieben mit und 31 % der Arbeitenden in Betrieben ohne Tarifvertrag bekommen ein aufgestocktes Kurzarbeitergeld (Hans-Böckler-Stiftung 2020, S. 1). Wer im Niedriglohnbereich arbeitet, kann damit kaum die laufenden Kosten bestreiten. 82 % der Befragten mit einem Haushaltsnettoeinkommen unter 1500 € machen sich Sorgen um ihre wirtschaftliche Zukunft (ebd., S. 2). Selbstständige, deren Gewerbe lahmgelegt ist, erleiden ein ähnliches Schicksal. Die Umstellung auf digitale Kommunikation ist ein weiterer Ungleichheitsverstärker. In den Schulen fallen diejenigen durch das Raster, die auf persönliche Kontakte und Hilfestellungen durch Bezugspersonen besonders angewiesen sind. Ungleichheitsverstärkend wirkt die Pandemie auch in scheinbar privilegierten Milieus. Weil der Nebenjob verloren geht, gerät das Studium in Gefahr. Homeoffice und digitale Kommunikation erweitern trotz mancher Annehmlichkeit auch den Zugriff auf die Privatsphäre. Wenn Schulen und Kitas geschlossen oder nicht voll funktionsfähig sind, wird die Kinderbetreuung zu einem schwer lösbaren Problem.

All das geschieht in reichen Ländern, die, wie Deutschland, noch immer über einigermaßen intakte soziale Sicherungssysteme verfügen. Wo wohlfahrtsstaatliche Netze nicht oder nur rudimentär vorhanden sind, fallen die Folgen von Pandemie und Rezession noch ungleich härter aus. Mit der Verlagerung des geografischen Zentrums der Pandemie in die USA und Teile des globalen Südens vollzieht sich zeitgleich eine Verschiebung auch der sozialen Brennpunkte der Seuche. Zunächst als Krankheit der Reichen klassifiziert, erfasst COVID-19 mehr und mehr die Elendszonen der Welt. Der Subkontinent Lateinamerika liefert Anschauungsunterricht. Am Beispiel von Guayaquil, einer Hafenstadt in Ecuador, hat der Philosoph Paul B. Preciado eindrucksvoll beschrieben, was in vielen Corona-Hotspots geschieht: „Es ist eine segregierte Stadt, die Armut ist überall sichtbar, es ist die Stadt, wo der Gegensatz von Zentrum und Peripherie am offensichtlichsten wird. Es gibt sehr viele wohnungslose Menschen […], sie schlafen im Zentrum auf der Straße, Menschen, die aus Venezuela gekommen sind, aber auch aus Guayaquil selbst, Sexarbeiterinnen oder Jugendliche, die Drogen konsumieren. Das Gesundheitssystem war schon vorher kollabiert. Das Virus hat all die Probleme verschärft, die schon vorher da waren“ (zit. nach Ruano 2020).

In vielen Ländern des Subkontinents sorgen prekäre Lebensverhältnisse, schlechte Ernährung, beengte Wohnverhältnisse und mangelhafte medizinische Versorgung für eine rasche Ausbreitung des Virus. Fahrlässiges Krisenmanagement wirkt als Beschleuniger. Selbst im verhältnismäßig entwickelten Argentinien gibt es begründete Ängste, dass mehr Menschen an den Folgen des Lockdowns sterben werden als an der Pandemie. COVID-19 bedeutet für viele sozialen Abstieg, Verlust der Wohnung und Hunger (vgl. Blecha 2020). Ähnlich verhält es sich in Chile, Brasilien, Peru und anderen lateinamerikanischen Staaten. Migranten, die in den Städten ihre Einnahmequelle verloren haben und sich wegen des zusammengebrochenen Transportsystems zu Fuß auf den Weg in die Heimat machen, sind der Seuche schutzlos ausgeliefert. Indigene Bevölkerungsgruppen, die, wie in Bolivien, von der Lebensmittelversorgung abgeschnitten werden, sehen sich ebenfalls existenziell bedroht (vgl. Clacso 2020a, 2020b). Ähnliche Muster können in zahlreichen weiteren Ländern des globalen Südens beobachtet werden. Gleich ob chinesische oder indische Wanderarbeiter, brasilianische Favelas oder südafrikanische Townships – stets stellt sich die Frage, ob die gesundheitlichen Risiken einen längeren Lockdown und dessen katastrophale Folgen überhaupt rechtfertigen können (vgl. CEPAL 2020).

### Die Pandemie als Entsolidarisierungstreiber

In den Elendszonen sowohl des Nordens als auch des Südens finden wir bestätigt, was für Pandemien schon immer galt. Die Ungleichheit nimmt zu, und sie wird vor allem jenen schaden, denen alsbald auch noch der Teller für die Suppe fehlen könnte. Deshalb ist unwahrscheinlich, dass sich der Vorrang von Gesundheit vor Wirtschaftsinteressen dauerhaft durchhalten lässt. Zunehmende Ungleichheit steigert hingegen die Gefahr von Entsolidarisierungen. Donald Trump und seine Regierung zeigen, wie sich ein solches Verhalten zwecks Machtsicherung gezielt schüren lässt. In den USA hat sich über viele Jahrzehnte hinweg eine ethnisch fragmentierte Unterklasse herausgebildet, deren Angehörige den Staat nur noch als repressive Instanz erleben. Die Unterklassenbildung erfolgt qua Kriminalisierung (Wacquant 2009). Binnen vierzig Jahren hat sich die Zahl der Gefängnisinsassen verfünffacht. Dabei handelt es sich überwiegend um Arme, die in schwarzen Communities leben; einer von neun jungen schwarzen Männern befindet sich in Haft: „Knapp 60 % derer, die keinen High-School-Abschluss erlangen, kommen mit etwa Mitte 30 ins Gefängnis.“ (Goffman 2015, S. 11 f.) Weil Abstandsregeln in Unterklassenmilieus schwer einzuhalten sind, ist die Zahl der Infizierten und Toten dort überdurchschnittlich hoch.

Das nutzt der US-Präsident für sein polarisierendes Krisenmanagement. Sobald klar war, dass die Seuche vor allem „people of colour“, Arme und Schutzlose dahinrafft, votierte Trump entschieden dafür, die Wirtschaft rasch wieder hochzufahren. Diese Klassenpolitik von oben samt ihrer rassistischen Konnotationen ist eine der Ursachen für jene Massenproteste, die nach dem gewaltsamen Tod George Floyds nahezu alle US-amerikanischen Großstädte erfassten. In Portland reagierte der US-Präsident gegen das Votum von Gouverneuren und Stadtregierungen mit dem Einsatz von Spezialtruppen. Brasiliens Bolsonaro-Regierung, die, von der hohen Zahl der Corona-Toten unbeeindruckt, an der Grippe-Legende festhält, verfährt nach einem ähnlichen Strickmuster. Auch dort spitzen sich gewaltträchtige Konflikte zwischen Regierungsgegnern und -anhängern zu. Wie schon früher organisieren Fußballfanclubs den Protest (vgl. Braga 2017; Ganter 2020).

Gewiss, Trump und Bolsonaro, aber auch Orban, Putin und andere autoritäre Herrscher verkörpern mehr als nur die Tendenz zu bonapartistischen Demokratien. Sie sind die neuen Bonapartes. Ihre Regimes verlieren jedoch an Zustimmung, denn aus der Perspektive von Gegenbewegungen schüren sie ein Verhalten einzelner und sozialer Gruppen, das in mancherlei Hinsicht die „Grausamkeit von Hunden“ noch übertrifft. Auf diese Weise trägt das autoritäre Führungspersonal dazu bei, dass, wie Madeleine Albright prognostiziert, im Zentrum der westlichen Welt die Gefahr eines neuen Faschismus entsteht, der vor allem als „Mittel zur Erringung von Macht und deren Erhalt“ (Albright 2018, S. 18) dient und von existenziellen Ängsten hervorgetrieben wird. Das Krisenmanagement der kontinentaleuropäischen EU-Staaten folgt, trotz der illiberalen Demokratien Polens und Ungarns, erkennbar anderen Präferenzen. Auf deutlich unterscheidbarem Level ist Entsolidarisierung gleichwohl auch in der EU bekannt. Weil Hilfsprogramme zunächst nur auf nationaler Ebene beschlossen wurden, fühlte sich das durch die Pandemie besonders gebeutelte Italien von Europa im Stich gelassen. In der Bevölkerung des Landes machen sich noch immer Verletzungen bemerkbar, deren Ursachen bis zur Krise von 2007–2009 zurückreichen. Damals hatte die Austeritätspolitik gerade im europäischen Süden zu tiefen Einschnitten in die Gesundheitssysteme geführt. In der Lombardei, anfangs das europäische Epizentrum der Pandemie, war die Privatisierung im Gesundheitssektor seitens der heutigen Lega Salvini Premier besonders radikal vorangetrieben worden. Von den 5060 gemeldeten Intensivbetten stellt der gehätschelte Privatsektor nicht einmal 8 %. Das ist einer der Gründe für die hohen Todesraten und das Triagieren von Ärzten, die zu entscheiden hatten, welche Notfälle sie dem sicheren Tod überlassen mussten (vgl. Böhme-Kuby 2020).

Zweifellos hat die Austeritätspolitik, die widerstrebenden Regierungen über den europäischen Rettungsschirm aufgezwungen wurde, die Spaltung der EU in Zentrum und Peripherie zusätzlich verstärkt. Um ein weiteres Auseinanderdriften zu vermeiden, hatten Italien und Frankreich vorgeschlagen, die Kosten der Krise teilweise über Corona-Bonds zu finanzieren. Die deutsche Ablehnung dieses Instruments, das eine gemeinsame Kreditaufnahme der EU-Staaten an den Finanzmärkten als Mittel der Rezessionsbewältigung ermöglicht hätte, ist kein Ausweis europäischer Solidarität. Immerhin wurde mit einem 750-Milliarden-Programm zum Wiederaufbau der Wirtschaft ein Kompromiss gefunden, der vorerst auch die Länder an der südlichen EU-Peripherie zufriedenstellt. Auf dem verminten Feld der Migrations- und Flüchtlingspolitik ist dergleichen dagegen nicht in Sicht. Hier bleibt Entsolidarisierung ein Charakteristikum europäischer Politik. Es grenzt an ein Wunder, dass die Flüchtlingscamps an den europäischen Außengrenzen von Ansteckungen zunächst einigermaßen verschont geblieben sind. Wenige Infizierte im Lager Moria bewirkten auf der Insel Lesbos dann jedoch eine soziale Explosion. Das niedergebrannte Camp und die nachfolgenden Auseinandersetzungen zwischen nunmehr obdachlosen Flüchtlingen, einer ursprünglich hilfsbereiten Bevölkerung und den Sicherheitskräften vor Augen, gleicht die Migrationspolitik der EU einer moralischen Bankrotterklärung. Die Folgen treffen wiederum hauptsächlich jene südeuropäischen Länder, die neben der Austeritätspolitik und der Corona-Pandemie zusätzlich die Hauptlast des inhumanen Dublin-Systems bei der Aufnahme von Fluchtmigranten zu tragen haben. Dass die italienische Regierung dennoch allen illegal im Land lebenden Migranten einen rechtlich kodifizierten Aufenthaltsstatus gewähren will, ist – auch wenn es um billige Arbeitskräfte auf den Tomatenplantagen gehen mag – eines der wenigen hoffnungsvollen Zeichen, das den politischen Handlungsspielraum deutlich macht, den alle EU-Mitgliedsstaaten nutzen könnten.^8^

## Umsteuern – aber wie, wohin und mit wem?

Halten wir fest: Die alten Bewältigungsmuster von Seuchen hinterlassen in der Gegenwart noch immer ihre Spuren. Die gesellschaftliche Widerstandsfähigkeit gegenüber der Pandemie hängt entscheidend vom jeweiligen Gesundheitssystem, der Verfügung über halbwegs krisenfeste soziale Netze, von wohlfahrtsstaatlichen Sicherungen und der Finanzkraft von Nationalstaaten ab. Wie unter einem Brennglas macht die Krankheit all jene Unsicherheiten und Ungleichheiten sichtbar, die in modernen kapitalistischen Gesellschaften seit langem (re)produziert werden. Privatisierungen und die finanzielle Ausblutung der Gesundheitssysteme haben die soziale Resilienz zusätzlich derart geschwächt, dass COVID-19 zu einer ernsten Bedrohung der ökonomischen Globalisierung werden konnte. Natürlich lassen sich, wie in jeder Krise, auch zahlreiche Beispiele für solidarisches Handeln und Gemeinsinn entdecken. Wer in häuslicher Quarantäne ist, sieht sich aus der Nachbarschaft gut versorgt. Ältere können sich mitunter vor Hilfsangeboten kaum retten. Universitäten richten Corona-Hilfsfonds für Studierende ein, Gewerkschaften und Betriebsräte sorgen für einen besseren Gesundheitsschutz in Unternehmen und die geltenden Abstandsregeln können Massenproteste gegen Rassismus, Polizeigewalt und wachsende Ungleichheit nicht verhindern. Aber genügt all das, um jene Nachhaltigkeitsrevolution in Gang zu setzen, nach der die Krise hinter der Pandemie so dringend verlangt?

### Degrowth by disaster

Der Rezessionsverlauf zeigt, dass entsprechende Weichenstellungen bitter nötig wären. Wie der Crash von 2007 bewirken Lockdown und Wirtschaftskrise „degrowth by disaster“ (Victor 2008). Tatsächlich haben eingeschränkte Mobilität und zeitweiliger Zusammenbruch der Industrie die Kohlendioxidemissionen in einem Maße reduziert, wie das seit Jahrzenten nicht mehr der Fall gewesen ist (IEA 2020). Doch mit der Wiederbelebung der Wirtschaft steigen die Emissionen rascher an als erwartet. Selbst wenn es bei dem für 2020 erwarteten CO_2_-Minus von 8 % bliebe, wäre der Gesamteffekt gering. Der menschengemachte Klimawandel ginge nahezu ungebremst weiter. Hinzu kommt, dass in Industriestaaten wie der Bundesrepublik neben dem Fahrrad vor allem der individuelle Pkw-Verkehr von den Corona-Regeln profitiert, während die Fahrgastzahlen bei der Bahn und im öffentlichen Nahverkehr in Stadt und Land um 70–80 % eingebrochen sind (Prätorius 2020). Offenbar steigert die Corona-Krise die Gefahr einer Schädigung von Sektoren, die für eine nachhaltige Verkehrswende unverzichtbar sind.

Harte Verteilungskämpfe, wie sie infolge hoher Verschuldung bei gleichzeitig sinkenden Steuereinnahmen allen Gesellschaften bevorstehen, könnten diesen Trend verstärken. In Deutschland plädieren die Wirtschaftsverbände bereits für rigide Sparmaßnahmen, die in erster Linie zulasten von Sozialausgaben gehen sollen.^9^ In anderen europäischen Staaten fallen Forderungen der Kapitalverbände noch erheblich radikaler aus. Deshalb ist es gut möglich, dass sich infolge der Pandemie in weit größerem Ausmaß ereignen wird, was in den deutschen Braunkohlerevieren bereits beobachtet werden kann. Dort haben sich die soziale und die ökologische Konfliktachse verselbständigt. Braunkohlebeschäftigte auf der einen, Klimabewegungen auf der anderen Seite setzen ihre Machtressourcen bevorzugt gegeneinander ein, obwohl sich alle um die Zukunft der Region sorgen (Roose 2020).

Der Konflikt um eine Kaufprämie für Pkw mit Verbrennungsmotor deutet an, dass im Wirtschöpfungssystem Automobil mit seinen 800.000 direkt sowie zwei Millionen indirekt Beschäftigten Ähnliches bevorstehen könnte. Einen Einbruch der Neuzulassungen um 43,5 % in Westeuropa, Milliardenverluste der Endhersteller und drohende Insolvenzen bei den Zulieferern vor Augen, priorisieren die Industriegewerkschaften Beschäftigungssicherheit. Ihre Führungsgruppen sind tief enttäuscht, weil sich, anders als 2009, ein Bonus für Pkw-Käufe nicht mehr durchsetzen ließ. Umgekehrt haben die Umweltverbände hohen Aufwand betrieben, um eine Abwrackprämie abzuwehren, ohne sich auf die Sicherheitsinteressen der Belegschaften überhaupt einzulassen. Obwohl beide Seiten die Klimaziele anerkennen, sind sie an Kooperation in Nachhaltigkeitsallianzen derzeit wenig interessiert (siehe die Fallstudien in Dörre et al. 2020). Radikalökologische Empfehlungen, die Wirtschaft im Abschaltmodus zu belassen (Latour 2020), bestärken die betroffenen Belegschaften in ihrer Neigung zu einer konservierenden Interessenpolitik. In Teilen versammeln sie sich hinter der Fahne populistischer Klimaleugner. Denn implizit legitimieren solche radikalökologischen Forderungen, was Unternehmen wie Airbus, Continental, Lufthansa, BMW, Scheffler, Kaufhof oder die Deutsche Bahn ohnehin beabsichtigen – einen drastischen Personalabbau und, so möglich, eine Verlagerung der Geschäfte in billigere Weltregionen.

### Der Corona-Staat

Unabhängig vom Verlauf der Transformationskonflikte wird künftig ein Staatsinterventionismus an Profil gewinnen, wie er ansatzweise bereits vor der Pandemie zu beobachten war. Gleich ob es um die Reorganisation von Wertschöpfungsketten, die Schaffung von Infrastruktur für E‑Mobilität, um die Digitalisierung oder die Vorsorge vor neuen Gesundheitsrisiken geht – der Staat wird mitmischen, andernfalls drohen Niederlagen in der imperialen Rivalität. Staatsinterventionismus allein ist aber kein Garant für Fortschritt in Sachen sozialer und ökologischer Nachhaltigkeit. Selbstverständlich ist der Staat im Kapitalismus kein bloßer Ausschuss herrschender Klassen. Er ist nicht homogener Akteur, sondern ein soziales Verhältnis, das sich in unterschiedlichen Staatsformen ausdrücken kann (Poulantzas 2002 [1978]). Mit Pierre Bourdieu (2014, S. 19) gesprochen, verkörpert der Staat das „Monopol der legitimen symbolischen Gewalt“. Zu seinen allgemeinsten Funktionen gehört „die Produktion und Kanonisierung sozialer Klassifikationen“ (ebd., S. 29). Indem sie klassifizierenden Maßstäben zu Verbindlichkeit verhelfen, wirken Staatsaktivitäten jederzeit auf alle gesellschaftlichen Subsysteme ein. Ob Staatshandeln gegenüber der Marktkoordination Vorteile mit sich bringt, hängt wesentlich davon ab, wie es sich zu demokratischer Willensbildung verhält.

Die Tendenz zu bonapartistischen Demokratien in Rechnung gestellt, wäre es geradezu fahrlässig, den Staat des Ausnahmezustands als Beweis für die Veränderbarkeit der Welt zu feiern. Dieser Staat reagiert auf eine medizinische Katastrophe, mit zunehmender Beherrschbarkeit der Pandemie verliert er jegliche Legitimität. Hält die Pandemie hingegen lange an, werden Abstandsgebote für große Bevölkerungsmehrheiten zu einer großen Last, weil sie auf radikale Entgesellschaftung und Entgemeinschaftung hinauslaufen. Alles, was dem Ausnahmestaat positiv zugeschrieben wird – Entschleunigung des Alltags, Konsumverzicht, Verkehrsvermeidung und Zeit für die Sorge um sich selbst –, ließe sich nach dem Abklingen der Pandemie nur noch auf freiwilliger Basis aufrechterhalten. Der erkennbare Drang zur Wiederherstellung einer Vor-Corona-Normalität lässt indes erahnen, wie wenig realitätstauglich derartige Erwartungshaltungen sind. Hoffnungen auf einen therapeutischen Effekt der Pandemie werden voraussichtlich auch deshalb enttäuscht, weil der intervenierende Wirtschaftsstaat einer Schrumpfung ökonomischer Aktivität direkt entgegenwirkt. Die Legitimität schuldenfinanzierter Wiederaufbauprogramme bemisst sich am Wachstumserfolg. Insofern ist der Corona-Staat ein Hybrid. Der Wirtschaftsstaat muss die Suppe auslöffeln, die ihm sein ungleicher Zwilling, der Ausnahmestaat, eingebrockt hat.

Die ökonomischen Staatsaktivitäten folgen einer Methode, die sich bereits während des globalen Finanzcrashs durchgesetzt hatte. Damals legte die Krise offen, „dass wir in einem Zeitalter nicht der staatlichen Zurückhaltung, sondern des ‚großen Regierens‘ lebten, einem Zeitalter […] eines Interventionismus, der in seiner Logik eher militärischen Operationen oder medizinischer Nothilfe glich als gesetzmäßiger Regierungsarbeit“ (Tooze 2018, S. 19). Im Corona-Staat setzt sich diese Tendenz überraschend fort. Überraschend, weil entgegen verbreiteter Erwartungen heute noch weit voluminösere Rettungspakete finanziell möglich und politisch durchsetzbar sind als während des Finanzcrashs. Doch nicht einmal die Dollar-Billionen, die dem Wiederaufbau der Weltwirtschaft dienen sollen, garantieren, dass die Wende zur Nachhaltigkeit tatsächlich gelingt. Geld ist reichlich vorhanden, doch es fehlt den Akteuren an sozialer Fantasie, an industrie- und dienstleistungspolitischem Know-how. Deshalb ist die Gefahr groß, dass die Milliarden versickern, ohne eine Nachhaltigkeitswende entscheidend voranzutreiben.

Starker Druck aus den Lagern des „Geistes von Porto Alegre“ könnte dazu beitragen, dass sich das ändert. Doch dem steht der Ausnahmestaat mit seinem strengen Regelwerk entgegen. Die Proteste der weltweiten Klimabewegungen hat er zunächst – fast – erstickt; er bleibt ein Staat, der innerhalb der demokratischen Verfassung und doch gegen dieselbe regiert. Demokratie benötigt Gegenöffentlichkeit, Opposition, Streit, Disput, Versammlungen, Demonstrationen und Streiks. Sie verkörpert daher das genaue Gegenteil des Ausnahmestaates. In einer globalisierten Welt kann freilich jede größere Naturkatastrophe, jede neue Seuche Notstandsmaßnahmen legitimieren. Ungebremster Klimawandel bedeutet schon jetzt eine Zunahme von Wetterextremen, Dürren, Waldbränden, Hunger, Fluchtbewegungen und daraus entstehende Kriegsgefahr. „Äußere Stöße“, die der Corona-Pandemie folgen, würden nicht nur neue Notstandsmaßnahmen provozieren, sondern die Rückkehr des autoritären, gewalttätigen Staates begünstigen. In Lateinamerika, aber auch in den USA und anderen Teilen der Welt, ist das bereits mehr als eine bloße Befürchtung. Setzte sich das autoritäre Lager des „Geistes von Davos“ durch, wandelten sich die modernen Kapitalismen tatsächlich zu „Katastrophengesellschaft[en]“ (Beck 1986, S. 105; Hervorh. weggel.), in denen der Ausnahmestaat über den eigentlichen Souverän, ein dann weitgehend entrechtetes Volk, dauerhaft herrscht.

### Für eine öffentliche Soziologie der Nachhaltigkeit

Fassen wir zusammen: Die Welt und vor allem die früh industrialisierten und die Schwellenländer durchlaufen eine epochale Zangenkrise. Fällt die Überwindung dieser Krise an sich schon schwer, versetzt COVID-19 dem Bemühen um Nachhaltigkeit einen zusätzlichen Stoß. Zwar überwindet der Corona-Staat das politische Interregnum der Zwischenkrisenperiode, doch das Geschehen verlagert sich von der ökologischen wieder stärker zur sozioökonomischen Konfliktachse. Es fehlt, was schon ein gutes Jahrzehnt zuvor nicht erkennbar war – eine glaubwürdige Alternative zum Bestehenden. Ein „climate turn“ der arbeitsorientierten Akteure und ein labour turn in den ökologischen Bewegungen als Voraussetzung von Nachhaltigkeitskoalitionen, die im sozial-ökologischen Transformationskonflikt aus der Zivilgesellschaft heraus wirkmächtig sind, werden von der Pandemie und deren Folgen zusätzlich erschwert.

Kann Soziologie mehr leisten, als solche Tendenzen zu beobachten und gelegentlich zu kommentieren? Vorab: Eine „public Sociology“, wie ich sie in Anlehnung an Michael Burawoy und den von ihm initiierten „Global Dialogue“ vertrete (Aulenbacher et al. 2017), war nie als Programmatik für das gesamte Fach gedacht. Anders als Kritiker gelegentlich vermuten (Neidhardt 2017), folgt öffentliche Soziologie keiner politischen Richtung. Dem Neutralitätsgebot empirischer Forschung ist sie genauso verpflichtet wie jede andere Sozialwissenschaft. „Falschgeldproduktion“, vor der Ralf Dahrendorf (1957, S. IIV) angesichts des ständigen Drucks vom „Gläubiger“ Öffentlichkeit auf stets lieferbereite wissenschaftliche „Schuldner“ warnte, muss sie, wie Wissenschaft überhaupt, tunlichst vermeiden. Wahlverwandtschaften mit einem im deutschsprachigen Raum nahezu unbekannten „Sociological Marxism“ (vgl. Burawoy 2003), die ich für attraktiv, andere hingegen für riskant halten, sind selten. Daran mag es liegen, dass mir die Erkenntnis, der Staat beruhe „eigentlich immer auf Macht“ und könne seine Aufgaben zeitweilig in allen Subsystemen gegebenenfalls „mit Gewalt durchsetzen“ (Nassehi 2020), wenig spektakulär erscheint. Ein Staat, der trotz unbezweifelbar komplexer Krisenphänomene in unterschiedliche Subsysteme interveniert, ist für erhebliche Teile der Weltbevölkerung alltäglich Realität.

Auch wenn man Burawoys Staatsferne nicht teilen mag, bleibt der Einfluss demokratischer Zivilgesellschaften auf lebenswichtige Entscheidungen dennoch ein Gradmesser für Emanzipation. Nachhaltigkeitsziele, auf die sich die Staatengemeinschaft verpflichtet hat, können als Maßstab dienen, anhand dessen sich politische Weichenstellungen von einer „komplexen Außenposition“ (Boltanski 2010, S. 26) aus beurteilen lassen. Mit ihrer Hilfe kann sich eine Soziologie der Nachhaltigkeit an der Suche nach Auswegen aus der – durch COVID-19 zusätzlich verkomplizierten – Zangenkrise beteiligen. Sicherlich ähnelt ein solches Vorhaben einer Quadratur des Kreises. Einerseits drängt die Zeit, weshalb Veränderungen auch von oben, mit Hilfe reformwilliger kapitalistischer Eliten und über Marktmechanismen durchzusetzen sind. Andererseits reicht die bloße Behandlung von Symptomen nicht aus, um die Krankheit der Zangenkrise nachhaltig zu besiegen. Sich dieses Dilemmas bewusst, kann öffentliche Soziologie doch mit einer „experimentellen Utopistik“ (Wallerstein 2002) operieren. Anhand von SDGs kann sie transparent machen, welche Veränderungen sich bewähren und was in Sackgassen endet. Einige Forschungsfelder drängen sich experimenteller Utopistik förmlich auf.

Dazu gehört zunächst die Seuchenprävention. Bisher setzten Hygienemaßnahmen in der Regel erst dann ein, wenn die Krankheit auftrat. Eine kleine Gruppe von Virologen hat stattdessen angeregt, ein globales Früherkennungssystem zu entwickeln, das Pandemien gar nicht erst entstehen lässt. Ein solches System wäre, das ist sein Pferdefuß, ausgesprochen teuer (Wolfe 2020, S. 273). Es müsste weltweit errichtet werden, um zu wirken, und würde daher die Prioritäten öffentlicher Haushalte dramatisch verändern. Groß angelegte Rüstungsprogramme ließen sich dann kaum mehr finanzieren. Seuchenprävention liefe somit auf tiefgreifende gesellschaftliche Veränderungen hinaus. Ob und wie sich ein solches System als öffentliches Gut realisieren lässt, ist daher auch eine soziologisch relevante Frage.

Ihre Beantwortung öffnet sogleich ein weiteres Feld. Allein mit marktwirtschaftlichen Mitteln sind krisenfeste Gesundheitssysteme nicht zu finanzieren. Gleiches gilt für Vorschläge, den Klimawandel hauptsächlich mit marktkonformen Instrumenten wie einem CO_2_-Preis zu bekämpfen (vgl. Leopoldina 2019, 2020). Länder wie die Schweiz, die eine Emissionssteuer samt Rückverteilungskomponente bereits eingeführt haben, zeigen, dass dergleichen für eine Nachhaltigkeitswende keineswegs ausreicht. Unter markwirtschaftlich-kapitalistischen Bedingungen böten nicht einmal handelbare individuelle CO_2_-Kontingente (von Weizsäcker 2020, S. 87) eine Nachhaltigkeitsgarantie. Würden sie doch, wie schon frühere Versuche des Emissionshandels, hochspekulativen Wetten auf die Zukunft Tür und Tor öffnen und so das Gegenteil ökologischer Nachhaltigkeit bewirken.

Mögliches Marktversagen vor Augen, stellt sich einer öffentlichen Soziologie die Frage, wie die Umstellung auf eine ressourcenschonende, kohlenstoffarme Produktion mit langlebigen Gütern zu bewerkstelligen ist. Nachhaltige Qualitätsproduktion bedeutet, weniger, dafür aber höherwertige Güter zu konsumieren. Entsprechende Weichenstellungen sind ohne den Bruch mit Produktionsabläufen, die primär von Märkten und Konsumenten her konzipiert werden, kaum vorstellbar. Der Übergang zu nachhaltiger Qualitätsproduktion wird wohl nur gelingen, wenn die Erzeugnisse einer solchen Produktionsweise trotz höherer Preise auch noch von den untersten Einkommensgruppen konsumiert werden können. Dergleichen ist ohne demokratische Rückverteilung zugunsten geringerer Einkommen ausgeschlossen.

Materielle Umverteilung reicht, auch wenn sie mit Arbeitszeitverkürzung verbunden wird, für eine Nachhaltigkeitsrevolution jedoch nicht aus. Schon jetzt eskalieren Transformationskonflikte vor allem an der ungleich verteilten Entscheidungsmacht über Investitionen und die stoffliche Beschaffenheit der Güterproduktion. In ökologischen Bewegungen und Sozialverbänden mehren sich Stimmen, die Weichenstellungen zugunsten einer nachhaltigen Gemeinwohlökonomie verlangen. Themen wie das einer Wirtschaftsdemokratie, in früheren Kontroversen noch mit Orchideenstatus versehen (vgl. Dörre et al. 2009, S. 301), stoßen mittlerweile nicht nur in gewerkschaftsnahen Kreisen (Urban 2018), sondern auch in einem Wissenschaftsspektrum auf Resonanz, das derartige Eingriffe in die Wirtschaft früher als Rückfall in vormoderne Entdifferenzierung kritisiert hätte (Herzog und Kuch 2020). So unterschiedlich sozial-ökologische Utopien auch ausfallen (vgl. Görgen und Wendt 2020) – sie alle eint der Gedanke, dass sich eine nachhaltige Gesellschaft nicht primär auf Konkurrenz, Gewinnmotiv und kapitalistischen Besitz gründen kann.

Bloße Appelle an kapitalistische Eliten, die Moderne neu zu erfinden, werden allerdings wenig fruchten. Mitunter scheint es, als begnüge sich manche sozialtheoretische Kritik damit, den Kapitalismus vor allem geistig zu überwinden. Intellektuell besiegt und als mentale „Schicksalsmacht“ in Frage gestellt, ist die widerborstige kapitalistische Realität selbst schuld, wenn sie sich den kritischen Vorgaben nicht fügt. Zur Wiedergeburt dieser neuen deutschen Ideologie, deren Protagonisten sich bevorzugt an das reformwillige Lager des „Geistes von Davos“ wenden, sollte sich eine öffentliche Soziologie der Nachhaltigkeit kritisch-konstruktiv verhalten. Ihr Ziel kann nur sein, den visionären Überschuss des „build back better“ zu erden – und zwar auch dann, wenn der Lockdown als willkommene Gelegenheit zu innerer Einkehr und Befreiung von Beschleunigungszwängen fehlinterpretiert wird.

Karl Marx benötigte empirische Analysen zur „Lage der arbeitenden Klassen in England“ (Engels 1972 [1845]), um über den Idealismus der Junghegelianer hinausgehen zu können. Empirisch fundierte Berichte zur Lage der beherrschten Klassen während und nach der Corona-Pandemie sind für eine experimentelle soziologische Utopistik ebenfalls ein Muss. Damit ist angesprochen, was öffentliche Soziologie sich einzugestehen hat. Institutionen, die die Lager des „Geistes von Porto Alegre“ mit den Reformern des „Geistes von Davos“ in einen produktiv-streitbaren Arbeitszusammenhang bringen könnten, existieren trotz mancher Lernfortschritte in den Klimabewegungen allenfalls im Larvenstadium. Sie finden sich in ausreichendem Maße weder in Parteien und Gewerkschaften noch in Staatsapparaten oder Nichtregierungsorganisationen. Deshalb kommt eine Nachhaltigkeitsrevolution vermutlich nicht ohne institutionelle Innovationen aus. Transformations- und Nachhaltigkeitsräte (Atkinson 2010; Dörre et al. 2020, S. 69, 300) könnten diese institutionelle Lücke füllen.

Die Größe der anstehenden Aufgaben vor Augen, gibt es für übertriebenen Optimismus jedoch keinen Anlass. „Durchkommen!“ lautet eine Devise, der sich viele verschrieben haben. Deshalb wäre es grundfalsch, dem Wünschbaren den Rang einer wahrscheinlichen Zukunft zu verleihen. Debatten um alternative Gesellschaftsmodelle sind sinnvoll, das waren sie auch schon vor der Pandemie. Doch nur Realitätssinn, gepaart mit der Skepsis des Verstandes, einem klaren Blick für Kräfteverhältnisse, für das Gangbare und das Erreichbare, kann Visionen nachhaltiger Gesellschaften den Status glaubwürdiger Alternativen verleihen. Anteilnahme am Schicksal all derer, die an COVID-19 erkranken, sterben oder wegen der Folgen der Pandemie in Not geraten, ist dafür unentbehrlich. Experimentierfreudigkeit mit wissenschaftlicher Expertise zu verbinden, um denen, die sonst unsichtbar blieben, eine Stimme zu geben, ist das mindeste, was eine öffentliche Soziologie der Nachhaltigkeit zu leisten hat. Dessen ungeachtet bleibt als vorläufiges Resümee: Die Kombination aus Pandemie, Rezession und Zangenkrise ist historisch einzigartig. Sie fügt sich nicht in gängige wissenschaftliche Krisendeutungen. Schon ihre Analyse verlangt nach einer kollektiven Kraftanstrengung, die Grenzziehungen zwischen den Sozial- und Naturwissenschaften systematisch überschreitet. Auch deshalb wirkt das stets wiederkehrende Stereotyp von der Krise als Chance, mitsamt der in ihr verborgenen Hoffnung auf eine „Sinnhaftigkeit der Zeit“ (Steil 1993, S. 11), besonders hohl. Der Bruch in den Gesellschaft-Natur-Beziehungen, wie er sich im Begriff des Anthropozäns oder, kontrastierend, des „Kapitalozäns“ (Moore 2015, S. 71) artikuliert, enthält zwei Botschaften. Die Menschheit kann mittels Überwindung hinderlicher Strukturen zur bewussten Hüterin der Natur werden, es liegt in ihrer Hand, verkrustete Machtverhältnisse zugunsten sozialer und ökologischer Nachhaltigkeit aufzubrechen. Sie kann, worauf der Nobelpreisträger Paul Crutzen verweist, das Menschenzeitalter aber auch beenden – durch Ökozid, einen verheerenden Atomschlag oder eine außer Kontrolle geratene Pandemie (Crutzen 2019, S. 173).

## Footnotes

1 Die Rede von einer Krisenmechanik war mir freilich immer suspekt – weil es in großen Krisen stets der sichtbaren Hand des Staates bedarf, um die Wachstumsdynamik wiederzubeleben (Dörre 2009, S. 43).

2 „Auf eine Formel gebracht: *Not ist hierarchisch, Smog ist demokratisch.*“ (Beck 1986, S. 48; Hervorh. i. Orig.).

3 Die „double internality“ (Moore 2015, S. 1), eine Bewegung des Kapitalismus durch Natur und von Natur durch den Kapitalismus, ändert nichts daran, dass die menschliche Natur analytisch von der außermenschlichen Natur zu trennen ist.

4 Die Spanische Grippe forderte mindestens 50 Mio. Menschenleben. Sie drang bis auf die entlegensten Südseeinseln vor und kostete Italien 1 %, Mexiko gar 4 % seiner Bevölkerung (Osterhammel 2009, S. 271).

5 Als Repulsion bezeichne ich nur solche Auswirkungen der Globalisierung, die in gewisser Weise auf das Gegenteil transnationaler Verflechtungen hinauslaufen.

6 Auch ohne Lockdown steckt Schwedens Wirtschaft wegen ihres hohen Internationalisierungsgrades und der verletzlichen Wertschöpfungsketten in einer tiefen Krise.

7 Schäuble ist freilich jener Politiker, dem der Historiker Adam Tooze persönlich eine Hauptverantwortung für das chaotische Management der zurückliegenden Eurokrise zuschreibt (Tooze 2018, S. 25).

8 Die Regelung wird allerdings nur von wenigen Fluchtmigranten in Anspruch genommen, weil sie sich in der Praxis als lebensfremd erweist (Manzi 2020).

9 Stopp für die Grundrente, die Eingrenzung sachgrundloser Befristung und die Rente mit 63, Rückabwicklung der paritätischen Finanzierung von Krankenversicherungsbeiträgen und Begrenzung der Leiharbeit sowie Einschränkung von Mitbestimmungsrechten lauten die Forderungen des für die Metallwirtschaft zuständigen Branchenverbandes (vgl. Gesamtmetall 2020).

## References

[CR1] Albright M (2018). Faschismus. Eine Warnung.

[CR2] Arendt H (2006). Elemente und Ursprünge totaler Herrschaft. Antisemitismus, Imperialismus, totale Herrschaft.

[CR3] Atkinson A (2018). Ungleichheit. Was wir dagegen tun können.

[CR4] Aulenbacher B, Burawoy M, Dörre K, Sittel J (2017). Öffentliche Soziologie – Wissenschaft im Dialog mit der Gesellschaft.

[CR5] Azzará S (2019). Rivolta populista e democrazia bonapartista postmoderna: un mio articolo su. Z. Zeitschrift Marxistische Erneuerung.

[CR7] Beck U (1986). Risikogesellschaft. Auf dem Weg in eine andere Moderne.

[CR6] Beck M, Stützle I (2018). Die neuen Bonapartisten. Mit Marx den Aufstieg von Trump & Co. verstehen.

[CR8] Blecha, L. (2020). Pragmatismus in Argentinien. *amerika21*.* Nachrichten und Analysen aus Lateinamerika*, 19. Juni 2020. https://amerika21.de/analyse/240702/corona-pragmatismus-argentinien. Zugegriffen: Juli 2020.

[CR11] Böhme-Kuby, S. (2020). SOS Italien. *Ossietzky. Zweiwochenschrift für Politik / Kultur / Wirtschaft*, *23* (7), 220–223.

[CR9] Boltanski L (2010). Soziologie und Sozialkritik.

[CR10] Bourdieu P (2014). Über den Staat. Vorlesungen am Collège de France, 1989–1992.

[CR12] Braga R (2017). A rebeldia do precaridado.

[CR13] Braudel F (1985). Der Alltag.

[CR14] Braudel F (1986). Aufbruch zur Weltwirtschaft.

[CR15] Brunkhorst H, Beck M, Stützle I (2018). Das revolutionäre Potenzial des Parlamentarismus. Überlegungen zum Bonapartismuskonzept von Marx. Die neuen Bonapartisten. Mit Marx den Aufstieg von Trump & Co. verstehen.

[CR16] Burawoy M (2003). For a sociological marxism: the complementary convergence of Antonio Gramsci and Karl Polanyi. Politics & Society.

[CR17] CEPAL (Comisión Económica para América Latinay el Caribe) (2020). Evitar una crisis alimentaria frente al COVID-19: Acciones urgentes contra el hambre. https://www.cepal.org/sites/default/files/presentation/files/version_final_200616_ppt_covid19-fao-cepal.pdf. Zugegriffen: Juli 2020.

[CR18] Clacso (2020a). Teoria & Cambio social. La crisis mundial del covid-19 (I). https://www.clacso.org/boletin-1-la-crisis-mundial-por-el-covid-19-del-grupo-de-trabajo-teoria-social-y-Neslwrealidad-latinoamericana/. Zugegriffen: Juli 2020.

[CR19] Clacso (2020b). Teoria & Cambio social. La crisis mundial del covid-19 (II). https://www.clacso.org/boletin-1-la-crisis-mundial-por-el-covid-19-del-grupo-de-trabajo-teoria-social-y-Neslwrealidad-latinoamericana/ Zugegriffen: Juli 2020.

[CR20] Climate Accountability Institute (2019). Press release on carbon majors update, 1965–2017, 9 October 2019. https://climateaccountability.org/pdf/CAI%20PressRelease%20Top20%20Oct19.pdf. Zugegriffen: Juli 2020.

[CR21] Crutzen PJ (2019). Das Anthropozän.

[CR22] Dahrendorf R (1957). Soziale Klassen und Klassenkonflikt in der industriellen Gesellschaft.

[CR23] Dörre K, Dörre K, Lessenich S, Rosa H (2009). Die neue Landnahme: Dynamiken und Grenzen des Finanzmarktkapitalismus. Soziologie – Kapitalismus – Kritik. Eine Debatte.

[CR24] Dörre K, Dörre K, Rosa H, Becker K, Bose S, Seyd B (2019). Risiko Kapitalismus. Landnahme, Zangenkrise, Nachhaltigkeitsrevolution. Große Transformation? Zur Zukunft moderner Gesellschaften.

[CR25] Dörre K, Bose S, Lütten J, Köster J (2018). Arbeiterbewegung von rechts? Motive und Grenzen einer imaginären Revolte. Berliner Journal für Soziologie.

[CR26] Dörre K, Holzschuh M, Köster J, Sittel J (2020). Abschied von Kohle und Auto? Sozial-ökologische Transformationskonflikte um Energie und Mobilität.

[CR27] Dörre K, Lessenich S, Rosa H (2009). Soziologie – Kapitalismus – Kritik. Eine Debatte.

[CR28] Engels F, Marx K, Engels F (1972). Die Lage der arbeitenden Klassen in England. Nach eigener Anschauung und authentischen Quellen. Werke (MEW).

[CR29] Federici S (2015). Caliban und die Hexe. Frauen, der Körper und die ursprüngliche Akkumulation.

[CR30] Fromm, T., & Hägler, M. (2020). Corona: Wo es der Wirtschaft besonders wehtut. *Süddeutsche Zeitung* vom 20. Juni 2020*.* https://www.sueddeutsche.de/wirtschaft/corona-schaeden-wo-es-der-wirtschaft-besonders-wehtut-1.4941749. Zugegriffen: Juli 2020.

[CR32] Fulcher J (2007). Kapitalismus.

[CR33] Galbraith JK (2008). Die Weltfinanzkrise – und was der neue US-Präsident tun sollte. Blätter für deutsche und internationale Politik.

[CR34] Gallagher, K., & Kozul-Wright, R. (2019). A new multilateralism for shared prosperity: Geneva principles for a global green new deal. https://unctad.org/en/pages/PublicationWebflyer.aspx?publicationid=2441. Zugegriffen: Juni 2020.

[CR35] Ganter, J. (2020). Brasilien im Katastrophenmodus. *Lateinamerika Nachrichten*, (552). https://lateinamerika-nachrichten.de/artikel/brasilien-im-katastrophenmodus/. Zugegriffen: Juli 2020.

[CR36] Gerhardt V, Crutzen PJ (2020). Die normative Wende im Anthropozän. Das Anthropozän: Schlüsseltexte des Nobelpreisträgers für das neue Erdzeitalter.

[CR31] Gesamtmetall (2020). Wiederhochfahren und Wiederherstellung. Vorschläge für die 2. und 3. Phase der Corona-Krise.

[CR38] Goffman A (2015). On the run. Die Kriminalisierung der Armen in Amerika.

[CR39] Goodhart D (2017). The road to somewhere: the populist revolt and the future of politics.

[CR37] Görgen B, Wendt B (2020). Sozial-ökologische Utopien. Diesseits oder jenseits von Wachstum und Kapitalismus.

[CR40] Gramsci A (1991). *Gefängnishefte. *Kritische Gesamtausgabe.

[CR41] Habermas, J. (2020). Jürgen Habermas über Corona: „So viel Wissen über unser Nichtwissen gab es noch nie“. *Frankfurter Rundschau* vom 10. April 2020. https://www.fr.de/kultur/gesellschaft/juergen-habermas-coronavirus-krise-covid19-interview-13642491.html. Zugegriffen: Juli 2020.

[CR42] Hall S, Hall S (2014). Nicos Poulantzas: Staatstheorie. Ausgewählte Schriften 5.

[CR43] Hans-Böckler-Stiftung (2020). *Böckler Impuls,* (12). https://www.boeckler.de/pdf/impuls_2020_12_gesamt.pdf. Zugegriffen: Aug. 2020.

[CR44] Herzog, L., & Kuch, H. (2020). Corona-Krise: Es ist Zeit für Wirtschaftsdemokratie. *Süddeutsche Zeitung* vom 17. Mai 2020. https://www.sueddeutsche.de/wirtschaft/wirtschaft-coronavirus-politik-neoliberal-demokratie-1.4910408. Zugegriffen: Juli 2020.

[CR45] IEA (International Energy Agency) (2020). Global energy review 2020. Paris: IEA. https://www.iea.org/reports/global-energy-review-2020. Zugriffen: Aug. 2020.

[CR46] Ifo Institut (2020). Konjunkturumfragen. Juni 2020. https://www.ifo.de/node/56337. Zugegriffen: Aug. 2020.

[CR49] ILO (International Labour Organisation) (2020). ILO Monitor, 2nd edition: Covid-19 and the world of work. Updated estimates and analysis. https://www.ilo.org/wcmsp5/groups/public/---dgreports/---dcomm/documents/briefingnote/wcms_740877.pdf. Zugegriffen: Juli 2020.

[CR47] IMF (International Monetary Fund) (2017). World economic outlook, April 2017. Gaining momentum? Washington. https://www.imf.org/en/Publications/WEO/Issues/2017/04/04/world-economic-outlook-april-2017. Zugegriffen: Juni 2020.

[CR48] IMF (International Monetary Fund) (2020). World economic outlook update, June 2020. A Crisis like no other, an uncertain recovery. Washington. https://www.imf.org/en/Publications/WEO/Issues/2020/06/24/WEOUpdateJune2020. Zugegriffen: Juni 2020.

[CR50] Jessop B (2018). Elective affinity or comprehensiv contradiction? Reflections on capitalism and democracy in the time of finance-dominated accumulation and austerity states. Berliner Journal für Soziologie.

[CR51] Kraushaar W (2012). Der Aufruhr der Ausgebildeten. Vom Arabischen Frühling zur Occupy-Bewegung.

[CR52] Latour, B. (2020). Welche Schutzmaßnahmen können wir uns vorstellen, damit wir nicht zum Produktionsmodell der Zeit vor der Krise zurückkehren? http://www.bruno-latour.fr/sites/default/files/downloads/P-202-AOC-ROSEN-ALLEMAND_0.pdf. Zugegriffen: Juli 2020.

[CR55] Leite, M. de P., Biavaschi, M., Salas, C., & Lima, J. (2020). *O trabalho em crise: flexibilidade e precariedades.* Resenha EBC Agência Brasil.

[CR53] Leopoldina, Nationale Akademie der Wissenschaften (2019). Klimaziele 2030. Wege zu einer nachhaltigen Reduktion der CO_2_-Emmissionen. https://www.leopoldina.org/uploads/tx_leopublication/2019_Stellungnahme_Klimaziele_2030_Final.pdf. Zugegriffen: Juli 2020.

[CR54] Leopoldina, Nationale Akademie der Wissenschaften (2020). Coronavirus-Pandemie – Die Krise nachhaltig überwinden. https://www.leopoldina.org/uploads/tx_leopublication/2020_04_13_Coronavirus-Pandemie-Die_Krise_nachhaltig_%C3%BCberwinden_final.pdf. Zugegriffen: Juli 2020.

[CR56] Maase K (2008). Die Schönheiten des Populären. Ästhetische Erfahrung der Gegenwart.

[CR57] Manzi, A. (2020): Apuliens Slum. *Der Freitag* Nr. 35 vom 27. August 2020.

[CR58] Marx K, Marx K, Engels F (1960). Der achtzehnte Brumaire des Louis Bonaparte. Werke (MEW).

[CR59] Moore JW (2015). Capitalism in the web of life.

[CR60] Nassehi, A. (2020). „Es ist eine digitalisierte Selbstbeobachtung der Gesellschaft“. *Frankfurter Rundschau* vom 24. April 2020. https://www.fr.de. https://www.fr.de/kultur/gesellschaft/armin-nassehi-eine-digitalisierte-selbstbeobachtung-gesellschaft-13715318.html. Zugegriffen: Juli 2020.

[CR61] Neidhardt F (2017). „Public Sociology“ – Burawoy-Hype und linkes Projekt. Berliner Journal für Soziologie.

[CR62] Osterhammel J (2009). *Die Verwandlung der Welt*.* Eine Geschichte des 19. Jahrhunderts*.

[CR64] Poulantzas N (2002). Der Staat, die Macht und der Sozialismus.

[CR65] Prätorius, G. (2020). Die Mobilitätswende im Lichte der Corona-Krise. *Verkehr und Technik*, *17*(6), 191–194.

[CR66] Roose, J. (2020). *Wirtschaft ist Heimat. Regionaler Strukturwandel in Biografien und Erwartungen der Bevölkerung.* Berlin: Konrad-Adenauer-Stiftung. https://www.kas.de/de/einzeltitel/-/content/wirtschaft-ist-heimat. Zugegriffen: Aug. 2020.

[CR67] Ruano, Y. O. (2020). Das Virus hat alle Probleme verschärft. Interview mit der Poetin und Frauenrechtlerin Yuliana Ortiz Ruano aus Guayaquil. *Lateinamerika Nachrichten*, (551). https://lateinamerika-nachrichten.de/artikel/das-virus-hat-alle-probleme-verschaerft/. Zugegriffen: Juli 2020.

[CR68] Schäuble, W. (2020). Aus eigener Stärke. *Frankfurter Allgemeine Zeitung* vom 6. Juli 2020. https://www.faz.net/aktuell/politik/inland/gastbeitrag-wolfgang-schaeuble-aus-eigener-staerke-16846887.html. Zugegriffen: Aug. 2020.

[CR555] Schmalz S, Sommer B (2019). Confronting Crisis and Precariousness. *Organized Labour and Social Unrest in the*.

[CR69] Statistisches Bundesamt (Destatis) (2020). Bruttoinlandsprodukt: Ausführliche Ergebnisse zur Wirtschaftsleistung im 2. Quartal 2020. https://www.destatis.de/Presse/Pressemitteilungen Zugegriffen: Aug. 2020.

[CR70] Steg J (2020). Normale Anomalie. Die Coronakrise als Zäsur und Chance. Blätter für deutsche und internationale Politik.

[CR71] Steil A (1993). Krisensemantik. Wissenssoziologische Untersuchungen zu einem Topos moderner Zeiterfahrung.

[CR72] Tooze A (2018). Crashed. Wie zehn Jahre Finanzkrise die Welt verändert haben.

[CR73] UN (United Nations) (2015). *Transforming our world: The 2030 Agenda for sustainable development*. https://sustainabledevelopment.un.org/content/documents/21252030AgendaforSustainableDevelopmentweb.pdf. Zugegriffen: Juli 2020.

[CR74] Urban H-J (2018). Ausbruch aus dem Gehäuse der Economic Governance. Überlegungen zu einer Soziologie der Wirtschaftsdemokratie in transformativer Absicht. Berliner Journal für Soziologie.

[CR75] Victor PA (2008). Managing without growth: slower by design, not disaster.

[CR76] Wacquant L, Castel R, Dörre K (2009). Die Wiederkehr des Verdrängten – Unruhen, „Rasse“ und soziale Spaltung in drei fortgeschrittenen Gesellschaften. Prekarität, Abstieg, Ausgrenzung. Die soziale Frage am Beginn des 21. Jahrhunderts.

[CR77] Wallerstein I (2002). Utopistik. Historische Alternativen des 21. Jahrhunderts.

[CR78] Wallerstein I, Wallerstein I, Collins R, Mann M, Derluguian G, Calhoun C (2014). Die strukturelle Krise oder Warum der Kapitalismus sich nicht mehr rentieren könnte. Stirbt der Kapitalismus? Fünf Szenarien für das 21. Jahrhundert.

[CR79] von Weizsäcker EU, Görgen B, Wendt B (2020). Eine spannende Reise zur Nachhaltigkeit. Naturkapitalismus und die neue Aufklärung. Sozial-ökologische Utopien. Diesseits oder jenseits von Wachstum und Kapitalismus.

[CR80] de Wilde P, Koopmans R, Merkel W, Strijbis O, Zürn M (2019). The struggle over borders: cosmopolitanism and communitarianism.

[CR81] Wolfe N (2020). Virus. Die Wiederkehr der Seuchen.

